# Factors associated with involuntary mental healthcare in New South Wales, Australia

**DOI:** 10.1192/bjo.2023.628

**Published:** 2024-03-04

**Authors:** Amy Corderoy, Matthew Michael Large, Christopher Ryan, Grant Sara

**Affiliations:** Discipline of Psychiatry and Mental Health, School of Clinical Medicine, Faculty of Medicine and Health, University of New South Wales, Australia; Discipline of Psychiatry, Centre for Values, Ethics, and the Law in Medicine, University of Sydney, Australia; Discipline of Psychiatry and Mental Health, School of Clinical Medicine, Faculty of Medicine and Health, University of New South Wales, Australia; Northern Clinical School, Faculty of Medicine and Health, University of Sydney, Australia; and InforMH, System Information and Analytics Branch, New South Wales Ministry of Health, St Leonards, New South Wales, Australia

**Keywords:** Involuntary, coercive, ethics, psychiatry and law, race

## Abstract

**Background:**

There is uncertainty about factors associated with involuntary in-patient psychiatric care. Understanding these factors would help in reducing coercion in psychiatry.

**Aims:**

To explore variables associated with involuntary care in the largest database of involuntary admissions published.

**Method:**

We identified 166 102 public mental health hospital admissions over 5 years in New South Wales, Australia. Demographic, clinical and episode-of-care variables were examined in an exploratory, multivariable logistic regression.

**Results:**

A total of 54% of eligible admissions included involuntary care. The strongest associations with involuntary care were referral from the legal system (odds ratio 4.98, 95% CI 4.61–5.38), and psychosis (odds ratio 4.48, 95% CI 4.31–4.64) or organic mental disorder (odds ratio 4.40, 95% CI 3.85–5.03). There were moderately strong associations between involuntary treatment and substance use disorder (odds ratio 2.68, 95% CI 2.56–2.81) or affective disorder (odds ratio 2.06, 95% CI 1.99–2.14); comorbid cannabis and amphetamine use disorders (odds ratio 1.65, 95% CI 1.57–1.74); unmarried status (odds ratio 1.62, 95% CI 1.49–1.76) and being born in Asia (odds ratio 1.42, 95% CI 1.35–1.50), Africa or the Middle East (odds ratio 1.32, 95% CI 1.24–1.40). Involuntary care was less likely for people aged >75 years (odds ratio 0.68, 95% CI 0.62–0.74), with comorbid personality disorder (odds ratio 0.90, 95% CI 0.87–0.94) or with private health insurance (odds ratio 0.89, 95% CI 0.86–0.93).

**Conclusions:**

This research strengthens the evidence linking diagnostic, socioeconomic and cultural factors to involuntary treatment. Targeted interventions are needed to reduce involuntary admissions in disadvantaged groups.

Despite community concerns and policies aimed at reducing the use of coercive mental healthcare,^1,2^ the incidence of involuntary psychiatric hospital admission in many high-income countries is increasing, and rates of involuntary care vary widely within and between countries.^3–6^ Variations in the use of involuntary care may reflect interactions between demographic, clinical and health system factors. Understanding factors associated with involuntary as opposed to voluntary care would help in developing strategies to decrease coercion in psychiatry,^6,7^ but there is uncertainty in the existing literature.

This study explores factors associated with involuntary compared with voluntary in-patient psychiatric treatment among all adults admitted over a 5-year period to public hospitals in the state of New South Wales (NSW), Australia. We examine the relationship between involuntary treatment and a range of covarying demographic, clinical and episode-of-care variables. This study contributes to evidence in several ways. We use a comprehensive, population-wide data-set, and include measures on a wide range of potential demographic and clinical predictors, including clinical status measures derived from the routinely collected Health of the Nation Outcome Scales (HoNOS) and Life Skills Profile (LSP). We provide data from the Asia-Pacific region, for which little data has been available in the past.

## Method

Data were obtained from the NSW Health admitted patient data collection. We conducted a cross-sectional study of all episodes of in-patient psychiatric care in government-funded (‘public’) hospital mental health services in NSW between 1 January 2016 and 29 July 2021.

### The cohort

In NSW, public in-patient mental health services have primary responsibility for acute, emergency and involuntary mental healthcare. In NSW in 2020, there were approximately 2600 mental health beds in more than 40 public hospitals, mostly in mental health units located within public general hospitals. These units provided approximately 38 000 episodes of in-patient hospital care.

In NSW, involuntary in-patient psychiatric care is regulated by the Mental Health Act 2007 (NSW). The Act restricts involuntary in-patient treatment to situations in which it is deemed that the patient or others either require protection from serious harm as a consequence of a ‘mental illness’ (defined as hallucinations, delusions, a serious disorder of thought form, severe disturbance of mood or sustained irrational behaviour indicating the presence of any of those), or require protection from serious physical harm caused by irrational behaviour.^8^ It must also be the case that no less restrictive means of providing safe and effective care is reasonably available.

We identified all episodes of in-patient care in public specialist mental health units in NSW between January 1 2016 and July 29 2021. To focus on involuntary acute psychiatric treatment in adults, we excluded: (a) episodes of care in child and adolescent units, older persons units, forensic, subacute and non-acute units; (b) people aged under 14 or over 100 years of age, who were admitted to adult or mixed mental health units; (c), episodes where people were admitted and discharged on the same day, because these are distorted by repeated admissions for day programmes and continuation electroconvulsive therapy; (d) episodes initiated by an administrative event (‘type change’) or within-hospital transfer and (e) episodes with no valid legal status recorded.

### Dependent variable: involuntary legal status

The dependent variable, involuntary legal status, classified each episode of hospital care as voluntary or involuntary. Episodes were defined as involuntary if the person's legal status was involuntary at any period during the episode, in line with Australian Institute of Health and Welfare reporting standards.^9^ Individual legal status codes were classified as either voluntary or involuntary with a reference table developed by NSW Health (Supplementary Table 1 available at https://doi.org/10.1192/bjo.2023.628).

### Independent variables

Factors potentially associated with involuntary legal status were identified *a priori* via a review of the primary literature,^10^ and by consensus judgement of the authors, and were organised into three groupings: (a) demographic factors, including age, gender, country of birth, language, rurality and socioeconomic status; (b) clinical factors, such as diagnosis, comorbidity, HoNOS scores and LSP scores; and (c) episode-of-care factors, including referral source and whether the person lived in the same service catchment area as the admitting hospital. NSW Health and Australian Bureau of Statistics reference tables were used to define service catchment areas of usual residence, regional groupings of country and language, and Socio-Economic Indexes for Areas scores, which rank areas within Australia according to relative socioeconomic disadvantage.

Primary and additional diagnoses were extracted for each episode of care. In NSW, diagnoses are coded by trained health information managers using the Australian Modification of the ICD-10 (ICD-10 AM),^11^ based on clinical notes and diagnoses entered by clinicians. Primary diagnoses were grouped into categories based on ICD code blocks. Substance-induced psychoses were counted as psychoses rather than substance use disorders. Comorbidity was identified when an episode had an additional diagnosis of a specific substance disorder (alcohol, cannabis, amphetamines), personality disorder or intellectual disability.

Routinely administered clinician-rated measures of the HoNOS, which include measures of aggression and self-harm,^12^ and the LSP^13^ were extracted, using the first measurement collected within each episode. For the HoNOS, adult (HoNOS) and older persons (HoNOS-65) were combined. Total and subscale scores were calculated according to Australia's national reporting guidelines.^14^ Total HoNOS scores were calculated where at least nine of the first 12 HoNOS items were validly answered, and divided into bands by using HoNOS score quartiles from national reference data for Australian mental healthcare admissions (https://www.amhocn.org/nocc-reporting/web-decision-support-tool), with bands defined as Q1 (total HoNOS score ≤9), Q2 (total HoNOS score 10–13), Q3 (total HoNOS score 14–18) and Q4 (total HoNOS score ≥19). HoNOS self-harm and aggression questions were scored as present (score of ≥2), absent (score of 0 or 1) or missing. The LSP score was dichotomised as 0–15 or ≥16, with higher scores representing greater need.

Scores were expressed categorically, with missing values treated as a separate category rather than excluded.

### Statistical analysis

Associations with the binary outcome variable (involuntary versus voluntary care) were examined with logistic regression. Independent variables were organised into three domains (described above): demographic factors, clinical factors and episode-of-care factors.

Selection of variables for inclusion in a final model then occurred in three stages. First, individual bivariate associations with involuntary as opposed to voluntary legal status were assessed for each variable. Second, a separate multivariable logistic regression was conducted for each of the three domains (demographic factors, clinical factors, episode-of-care factors). Individual variables with significant bivariate associations (*P* < 0.05) were entered into a multivariable model for each category, which was simplified after testing for multicollinearity by examination of condition indices. Third, all variables from the three domain-specific multivariable models were combined into a single multivariable logistic regression including demographic, clinical and episode-of-care factors, which was further simplified after testing for multicollinearity. The strength of the associations were classified as strong (odds ratio of >5 or <0.2), moderate (odds ratio of 1.5–5 or 0.20–0.67) or weak (odds ratio of 1–1.5 or 0.68–0.99), according to effect size equivalents.^15^

Statistical analyses were conducted in Stata version 15.1 for Windows, using Stata's ‘logistic’ procedure for all regressions. Some people had multiple admissions recorded in our data-set, and observations were likely to be correlated between those repeated admissions. Therefore, standard errors were estimated after clustering admissions within individuals (the ‘VCE-cluster’ option within Stata's ‘logistic’ procedure) by using a state-wide unique person identifier.

### Effects of COVID-19

In much of 2020 and all of 2021, service delivery in NSW hospitals was affected by the COVID-19 pandemic. A sensitivity analysis was therefore conducted after excluding episodes with a date of admission on or after 1 January 2020.

### Ethical approval

The authors assert that all procedures contributing to this work comply with the ethical standards of the relevant national and institutional committees on human experimentation and with the Helsinki Declaration of 1975, as revised in 2008. Approval for this project, including waiver of consent for data linkage and use, was granted by the NSW Population and Health Services Research Ethics Committee (approval numbers 2018/HRE0404 and HREC/18/CIPHS/18).

## Results

During the study period, there were 172 712 in-scope admissions to NSW mental health units. Of these, 6606 (3.8%) did not have a valid legal status recorded and were excluded from analysis (Supplementary Table 2). Episodes with missing legal status shared characteristics with voluntary episodes; being more likely in females and people aged under 24 years; being brief (more than half were of 3 days duration or less); with primary diagnoses of anxiety, adjustment disorder or injury and poisoning; having low HoNOS aggression scores; having private health insurance; living in less disadvantaged regions; being referred via the emergency department and living locally to the admitting service. They were less likely to have a primary diagnosis of psychosis, comorbid cannabis or amphetamine use. After exclusion of these episodes, there were 166 106 eligible hospital admissions, of which 89 695 (54%) included at least one day of involuntary care (see Table 1).

**Table 1:** Descriptive statistics for episodes of mental healthcare in New South Wales hospitals, January 2016 to July 2021, showing variables in demographic, clinical and episode-of-care domains

		Total cohort	Legal status	
Total		*n* (% of category)	Involuntary *n* (% of row)	Voluntary *n* (% of row)
Episodes of care		166 102 (100%)	89 994 (54%)	76 108 (46%)
Demographics				
Gender	Male	88 522 (53%)	51 127 (58%)	37 395 (42%)
	Female	77 507 (47%)	38 846 (50%)	38 661 (50%)
	Other	73 (0%)	21 (29%)	52 (71%)
Age group, years	14–17	10 010 (6%)	3722 (37%)	6288 (63%)
	18–24	27 324 (16%)	14 485 (53%)	12 839 (47%)
	25–35	38 895 (23%)	22 579 (58%)	16 316 (42%)
	35–45	36 841 (22%)	21 098 (57%)	15 743 (43%)
	45–55	28 719 (17%)	15 684 (55%)	13 035 (45%)
	55–65	14 690 (9%)	7987 (54%)	6703 (46%)
	65–75	6535 (4%)	3133 (48%)	3402 (52%)
	≥75	3092 (2%)	1309 (42%)	1783 (58%)
Country of birth	Africa and the Middle East	5921 (4%)	3608 (61%)	2313 (39%)
	Asia	10 408 (6%)	6405 (62%)	4003 (38%)
	Australia	134 050 (81%)	71 436 (53%)	62 614 (47%)
	Europe	3929 (2%)	2197 (56%)	1732 (44%)
	New Zealand and Pacific	4634 (3%)	2753 (59%)	1881 (41%)
	Other or unknown	2005 (1%)	1178 (59%)	827 (41%)
	UK, Ireland, USA or Canada	5159 (3%)	2420 (47%)	2739 (53%)
Language	Not English	8377 (5%)	5170 (62%)	3207 (38%)
	English	157 729 (95%)	84 827 (54%)	72 902 (46%)
Marital status	Married	30 134 (18%)	14 982 (50%)	15 152 (50%)
	Unknown	3546 (2%)	2483 (70%)	1063 (30%)
	Not married	132 426 (80%)	72 532 (55%)	59 894 (45%)
Employment	Employed	2772 (2%)	1011 (36%)	1761 (64%)
	Student	1968 (1%)	555 (28%)	1413 (72%)
	Unknown	149 707 (90%)	82 308 (55%)	67 399 (45%)
	Not employed	11 659 (7%)	6123 (53%)	5536 (47%)
Homeless	Yes	6097 (4%)	3436 (56%)	2661 (44%)
	No	160 009 (96%)	86 561 (54%)	73 448 (46%)
Socioeconomic disadvantage	Least (quintile 1–3)	89 416 (54%)	46 378 (52%)	43 038 (48%)
	Most (quintile 4–5)	60 400 (36%)	34 170 (57%)	26 230 (43%)
	Unknown	16 290 (10%)	9449 (58%)	6841 (42%)
Rurality	Major cities	76 741 (46%)	41 266 (54%)	35 475 (46%)
	Inner regional	53 871 (32%)	29 190 (54%)	24 681 (46%)
	Outer and remote	19 204 (12%)	10 092 (53%)	9112 (47%)
	Unknown	16 290 (10%)	9449 (58%)	6841 (42%)
Clinical				
Primary diagnosis	Affective	32 018 (19%)	15 672 (49%)	16 346 (51%)
	Anxiety and adjustment	24 897 (15%)	8281 (33%)	16 616 (67%)
	Eating disorder	653 (0%)	276 (42%)	377 (58%)
	Injury and poisoning	18 988 (11%)	8060 (42%)	10 928 (58%)
	Non-mental health	6928 (4%)	3702 (53%)	3226 (47%)
	Organic mental health disorder	1155 (1%)	774 (67%)	381 (33%)
	Other mental health	14 517 (9%)	6500 (45%)	8017 (55%)
	Psychosis	44 664 (27%)	32 009 (72%)	12 655 (28%)
	Substance use	22 286 (13%)	14 723 (66%)	7563 (34%)
Comorbidity	Alcohol	26 580 (16%)	14 097 (53%)	12 483 (47%)
	Cannabis	24 085 (15%)	16 183 (67%)	7902 (33%)
	Amphetamines	24 636 (15%)	17 116 (69%)	7520 (31%)
	No cannabis or amphetamines	127 272 (77%)	63 741 (50%)	63 531 (50%)
	Cannabis only	14 198 (9%)	9140 (64%)	5058 (36%)
	Cannabis and amphetamines	9887 (6%)	7043 (71%)	2844 (29%)
	Amphetamines only	14 749 (9%)	10 073 (68%)	4676 (32%)
	Personality disorder	27 609 (17%)	13 133 (48%)	14 476 (52%)
	Intellectual disability	4005 (2%)	2292 (57%)	1713 (43%)
Aggression (HoNOS)	No	72 647 (44%)	36 441 (50%)	36 206 (50%)
	Yes	31 600 (19%)	23 858 (76%)	7742 (25%)
	Unknown	61 859 (37%)	29 698 (48%)	32 161 (52%)
Self-harm (HoNOS)	No	70 698 (43%)	42 993 (61%)	27 705 (39%)
	Yes	33 549 (20%)	17 306 (52%)	16 243 (48%)
	Unknown	61 859 (37%)	29 698 (48%)	32 161 (52%)
HoNOS score	No HoNOS	61 859 (37%)	29 698 (48%)	32 161 (52%)
	Quintile 1 (Low)	42 885 (26%)	22 465 (52%)	20 420 (48%)
	Quintile 2	22 332 (13%)	12 799 (57%)	9533 (43%)
	Quintile 3	18 700 (11%)	11 378 (61%)	7322 (39%)
	Quintile 4 (High)	20 330 (12%)	13 657 (67%)	6673 (33%)
LSP	LSP High	2133 (1%)	1553 (73%)	580 (27%)
	LSP Low	1713 (1%)	832 (49%)	881 (51%)
	No LSP	162 260 (98%)	87 612 (54%)	74 648 (46%)
Episode of care				
Insurance status	None	144 041 (87%)	78 813 (55%)	65 228 (45%)
	Private	11 723 (7%)	5414 (46%)	6309 (54%)
	Unknown	10 342 (6%)	5770 (56%)	4572 (44%)
Source of referral	Community team or out-patient	16 409 (10%)	6361 (39%)	10 048 (61%)
	Crisis team	4651 (3%)	2558 (55%)	2093 (45%)
	Emergency department	81 507 (49%)	43 201 (53%)	38 306 (47%)
	Legal	5943 (4%)	5067 (85%)	876 (15%)
	Other hospital	35 416 (21%)	22 818 (64%)	12 598 (36%)
	Self or family	8196 (5%)	1833 (22%)	6363 (78%)
	Unknown and other	13 984 (8%)	8159 (58%)	5825 (42%)
Medicare eligible	No	1845 (1%)	1184 (64%)	661 (36%)
	Yes	164 261 (99%)	88 813 (54%)	75 448 (46%)
Emergency status	Emergency	138 744 (84%)	75 129 (54%)	63 615 (46%)
	Other/unknown	16 932 (10%)	10 592 (63%)	6340 (37%)
	Planned	10 430 (6%)	4276 (41%)	6154 (59%)
Catchment group	Interstate	3411 (2%)	2163 (63%)	1248 (37%)
	No fixed address/unknown	12 879 (8%)	7286 (57%)	5593 (43%)
	Other local health district	30 654 (18%)	15 935 (52%)	14 719 (48%)
	Same local health district	21 540 (13%)	11 842 (55%)	9698 (45%)
	Same catchment	97 622 (59%)	52 771 (54%)	44 851 (46%)
Voluntary unit	No	163 629 (99%)	89 943 (55%)	73 686 (45%)
	Yes	2477 (1%)	54 (2%)	2423 (98%)

HoNOS, Health of the Nation Outcome Scales; LSP, Life Skills Profile.

In this exploratory analysis, multiple demographic, clinical and episode-related variables were associated with involuntary as opposed to voluntary care in both bivariate and within-domain multivariable models (Table 2). Demographic factors were weakly to moderately associated with involuntary status on univariate analysis, including being male and not married. Higher rates of involuntary treatment were found in people aged 25–45 years; people living in more disadvantaged regions; those speaking a language other than English at home and people born outside Australia, the UK, Ireland, the USA or Canada.

**Table 2:** Associations between involuntary care and variables in the demographic, clinical and episode-of-care domains

		Bivariate		Multivariable within domain	
		Odds ratio	(95% CI)	Odds ratio	(95% CI)
Demographic					
Gender	Male	1.00	−	1.00	−
	Female	0.73	(0.72–0.75)***	0.78	(0.77–0.80)***
	Other	0.30	(0.18–0.49)***	0.35	(0.21–0.58)***
Age group, years	14–17	0.44	(0.42–0.46)***	0.47	(0.44–0.49)***
	18–24	0.84	(0.82–0.87)***	0.84	(0.81–0.86)***
	25–35	1.03	(1.00–1.06)*	1.02	(0.99–1.05)
	35–45	1.00	−	1.00	−
	45–55	0.90	(0.87–0.93)***	0.92	(0.89–0.95)***
	55–65	0.89	(0.86–0.92)***	0.91	(0.88–0.95)***
	65–75	0.69	(0.65–0.72)***	0.74	(0.70–0.78)***
	≥75	0.55	(0.51–0.59)***	0.61	(0.56–0.66)***
Country of birth	Africa and the Middle East	1.37	(1.30–1.44)***	1.26	(1.20–1.34)***
	Asia	1.40	(1.35–1.46)***	1.36	(1.30–1.42)***
	Australia	1.00	–	1.00	–
	Europe	1.11	(1.04–1.19)**	1.18	(1.10–1.26)***
	New Zealand and Pacific	1.28	(1.21–1.36)***	1.25	(1.18–1.33)***
	Other or unknown	1.25	(1.14–1.37)***	1.12	(1.03–1.23)*
	UK, Ireland, USA and Canada	0.77	(0.73–0.82)***	0.83	(0.78–0.88)***
Language	Not English	1.39	(1.32–1.45)***	1.13	(1.08–1.20)***
	English	1.00	−	1.00	−
Marital status	Married	1.00	−	1.00	−
	Unknown	1.22	(1.19–1.26)***	1.28	(1.25–1.31)***
	Not married	2.36	(2.19–2.55)***	2.21	(2.05–2.39)***
Employment	Employed	1.00	−		
	Student	0.68	(0.60–0.78)***		
	Unknown	2.13	(1.97–2.30)***		
	Not employed	1.93	(1.77–2.10)***		
Homeless	Yes	1.10	(1.04–1.15)**	0.98	(0.93–1.03)
	No	1.00	−	1.00	−
Socioeconomic disadvantage	Least (quintile 1–3)	1.00	−	1.00	−
	Most (quintile 4–5)	1.21	(1.18–1.23)***	1.20	(1.18–1.23)***
	Unknown	1.28	(1.24–1.33)***	1.14	(1.10–1.18)***
Rurality	Major cities	1.00	−		
	Inner regional	1.02	(0.99–1.04)		
	Outer and remote	0.95	(0.92–0.98)**		
	Unknown	1.19	(1.15–1.23)***		
Clinical					
Primary diagnosis	Affective	1.92	(1.86–1.99)***	1.90	(1.83–1.96)***
	Anxiety and adjustment	1.00	−	1.00	−
	Eating disorder	1.47	(1.25–1.72)***	1.64	(1.40–1.92)***
	Injury and poisoning	1.48	(1.42–1.54)***	1.53	(1.47–1.59)***
	Non-mental health	2.30	(2.18–2.43)***	2.44	(2.31–2.58)***
	Organic mental health disorder	4.08	(3.60–4.62)***	4.28	(3.77–4.86)***
	Other mental health	1.63	(1.56–1.70)***	1.61	(1.53–1.69)***
	Psychosis	5.08	(4.91–5.25)***	4.42	(4.27–4.57)***
	Substance use	3.91	(3.76–4.06)***	2.70	(2.59–2.82)***
Comorbidity	Alcohol	0.95	(0.92–0.97)***	0.00	
	Cannabis	1.89	(1.84–1.95)***		
	Amphetamines	2.14	(2.08–2.21)***		
	No cannabis or amphetamines	1.00	−	1.00	−
	Cannabis only	1.80	(1.74–1.87)***	1.56	(1.50–1.62)***
	Cannabis and amphetamines	2.47	(2.36–2.58)***	1.79	(1.70–1.87)***
	Amphetamines only	2.15	(2.07–2.23)***	1.55	(1.49–1.62)***
	Personality disorder	0.73	(0.71–0.75)***	0.91	(0.88–0.94)***
	Intellectual disability	1.14	(1.07–1.21)***	1.24	(1.15–1.32)***
Aggression (HoNOS)	No	1.00	−	1.00	−
	Yes	3.06	(2.97–3.15)***	2.59	(2.51–2.67)***
	Unknown	0.92	(0.90–0.94)***	0.96	(0.94–0.98)***
Self-harm (HoNOS)	No	1.00	−		
	Yes	0.69	(0.67–0.70)***		
	Unknown	0.60	(0.58–0.61)***		
HoNOS score	No HoNOS	1.00	–		
	Quintile 1 (low)	1.19	(1.16–1.22)***		
	Quintile 2	1.45	(1.41–1.50)***		
	Quintile 3	1.68	(1.63–1.74)***		
	Quintile 4 (high)	2.22	(2.14–2.29)***		
LSP	LSP high	2.84	(2.48–3.24)***	2.06	(1.79–2.37)***
	LSP low	1.00	−	1.00	−
	No LSP	1.24	(1.13–1.37)***	1.26	(1.14–1.40)***
Episode of care					
Insurance status	None	1.00	−	1.00	−
	Private	0.71	(0.68–0.74)***	0.71	(0.68–0.74)***
	Unknown	1.04	(1.00–1.09)*	1.01	(0.97–1.05)0
Source of referral	Community team or out-patient	0.56	(0.54–0.58)***	0.61	(0.59–0.63)***
	Crisis team	1.08	(1.02–1.15)*	1.13	(1.06–1.20)***
	Emergency department	1.00	−	1.00	−
	Legal	5.13	(4.77–5.52)***	5.02	(4.67–5.40)***
	Other hospital	1.61	(1.57–1.65)***	1.65	(1.61–1.69)***
	Self or family	0.26	(0.24–0.27)***	0.25	(0.24–0.27)***
	Unknown and other	1.24	(1.20–1.29)***	1.28	(1.23–1.32)***
Medicare eligible	No	1.52	(1.38–1.67)***		
	Yes	1.00	−	1.00	−
Emergency status	Emergency	1.00	−	1.00	−
	Other/unknown	1.41	(1.37–1.46)***	0.00	
	Planned	0.59	(0.57–0.61)***	0.00	
Catchment group	Interstate	1.47	(1.37–1.58)***	1.44	(1.34–1.55)***
	No fixed address/unknown	1.11	(1.07–1.15)***	1.03	(0.99–1.07)
	Other local health district	0.92	(0.90–0.94)***	0.90	(0.87–0.92)***
	Same local health district	1.04	(1.01–1.07)*	0.95	(0.92–0.98)**
	Same catchment	1.00	−		
Voluntary unit	No	1.00	−		
	Yes	0.02	(0.01–0.02)***	0.02	(0.01–0.02)***

Bivariate and multivariable associations using logistic regression separately within each domain. HoNOS, Health of the Nation Outcome Scales; LSP, Life Skills Profile.

* *P* < 0.05, ** *P* < 0.005, *** *P* < 0.0005.

Some of the strongest associations with involuntary as opposed to voluntary legal status were diagnostic, including diagnoses of psychosis (univariate odds ratio 5.08, 95% CI 4.91–5.25) or organic mental disorder (univariate odds ratio 4.08, 95% CI 3.60–4.62). There were moderate associations between involuntary care and primary diagnoses of affective disorders, or substance use disorders and comorbid diagnoses of intellectual disability, cannabis or amphetamine use disorders. Comorbid personality disorders were weakly associated with voluntary status.

Higher overall HoNOS scores and aggression subscale scores were also moderately associated with involuntary care, as were higher (more impaired) scores on the LSP. In contrast, high HoNOS self-harm scores were associated with reduced odds of involuntary care.

Significant episode-of-care factors included a strong association with referral from legal services (univariate odds ratio 5.13, 95% CI 4.77–5.52), moderate association with referral from other hospitals and weak association with referral from crisis teams and being an interstate visitor. It was less likely in planned admissions, people with private health insurance and, predictably, admissions to designated voluntary units.

In the final multivariable model combining all three domains (Table 3), the strongest independent associations with involuntary treatment were found with diagnosis of psychosis (odds ratio 4.48, 95% CI 4.31–4.64) and organic mental health condition (odds ratio 4.40, 95% CI 3.85–5.03), and referral to hospital via legal means (odds ratio 4.98, 95% CI 4.61–5.38).

**Table 3:** Associations between involuntary care and variables in the demographic, clinical and episode-of-care domains

		Multivariable	
		Odds ratio	(95% CI)
Gender	Male	1.00	–
	Female	0.96	(0.94–0.98)***
	Other	0.58	(0.34–0.99)*
Age group, years	14–17	0.75	(0.71–0.79)***
	18–24	1.07	(1.03–1.11)***
	25–35	1.08	(1.04–1.11)***
	35–45	1.00	−
	45–55	0.98	(0.95–1.01)
	55–65	1.00	(0.96–1.05)
	65–75	0.84	(0.79–0.89)***
	≥75	0.68	(0.62–0.74)***
Country of birth	Africa and the Middle East	1.32	(1.24–1.40)***
	Asia	1.42	(1.35–1.50)***
	Australia	1.00	−
	Europe	1.18	(1.10–1.27)***
	New Zealand and Pacific	1.18	(1.10–1.26)***
	Other or unknown	1.18	(1.07–1.31)**
	UK, Ireland, USA, Canada	0.93	(0.88–0.99)*
Language	Not English	1.11	(1.05–1.18)***
	English		
Marital status	Married	1.00	−
	Unknown	1.00	(0.98–1.03)
	Not married	1.62	(1.49–1.76)***
Employment	Employed	1.00	−
	Student	0.71	(0.62–0.82)***
	Unknown	1.32	(1.20–1.44)***
	Not employed	1.18	(1.07–1.30)**
Homeless	Yes	0.93	(0.88–0.99)*
	No	1.00	−
Primary diagnosis	Affective	2.06	(1.99–2.14)***
	Anxiety and adjustment	1.00	−
	Eating disorder	1.98	(1.68–2.34)***
	Injury and poisoning	1.57	(1.51–1.64)***
	Non-mental health	2.39	(2.25–2.53)***
	Organic mental health disorder	4.40	(3.85–5.03)***
	Other mental health	1.57	(1.49–1.65)***
	Psychosis	4.48	(4.31–4.64)***
	Substance use	2.68	(2.56–2.81)***
Comorbidity	Alcohol	0.92	(0.89–0.94)***
	Cannabis		
	Amphetamines		
	No cannabis or amphetamines	1.00	−
	Cannabis only	1.49	(1.43–1.56)***
	Cannabis and amphetamines	1.65	(1.57–1.74)***
	Amphetamines only	1.45	(1.39–1.51)***
	Personality disorder	0.90	(0.87–0.94)***
	Intellectual disability	1.19	(1.11–1.27)***
Aggression (HoNOS)	No	1.00	−
	Yes	2.53	(2.45–2.61)***
	Unknown	1.06	(1.03–1.09)***
LSP	LSP High	1.98	(1.71–2.29)***
	LSP Low	1.00	−
	No LSP	1.23	(1.11–1.37)***
Insurance status	None	1.00	−
	Private	0.89	(0.86–0.93)***
	Unknown	0.97	(0.93–1.02)
Source of referral	Community health centre and out-patient	0.68	(0.65–0.70)***
	Crisis team	1.00	(0.93–1.07)
	Emergency department	1.00	−
	Legal	4.98	(4.61–5.38)***
	Other hospital	1.87	(1.81–1.94)***
	Self or family	0.26	(0.25–0.28)***
	Unknown and other	1.30	(1.25–1.36)***
Emergency status	Emergency	1.00	−
	Other/unknown	0.85	(0.81–0.89)***
	Planned	0.62	(0.59–0.65)***
Catchment group	Interstate	1.47	(1.35–1.61)***
	No fixed Address/unknown	0.00	
	Other local health district	0.93	(0.90–0.96)***
	Same local health district	1.01	(0.98–1.04)
Voluntary unit	No		
	Yes	0.02	(0.02–0.03)***

Multivariable associations using logistic regression across all three domains. HoNOS, Health of the Nation Outcome Scales; LSP, Life Skills Profile.

* *P* < 0.05, **P < 0.005, ***P < 0.0005.

Other moderate associations were found with primary diagnoses of affective disorder, substance use disorder, eating disorder or self-injury and poisoning, as well as with high HoNOS aggression scores and low LSP scores. Amphetamine and cannabis disorders were collapsed in the multivariable model because of substantial collinearity, with the highest odds of involuntary treatment seen in episodes with both cannabis and amphetamine diagnoses. Among person factors, being unmarried was moderately associated with involuntary status.

Finally, a number of weaker associations were found across multiple domains, including country of birth, language spoken at home, employment status, private health insurance ownership, age and comorbid diagnoses.

No significant difference was found in sensitivity analysis when data from the COVID-19 period was excluded (Supplementary Table 3, (a)–(c)).

## Discussion

The chance of a person receiving involuntary care likely reflects a complex interplay between factors relating to the individual, their illness and the provider or episode of care.

We report an exploratory analysis factors associated with involuntary admission in a large sample of more than 166 000 admissions, of which 54% were involuntary, more than any primary study reported in the most recent meta-analysis.^10^

The strongest associations in our data were psychosis and organic mental health diagnoses, and being brought to hospital via legal means, likely reflecting the requirements of the NSW Mental Health Act.

However, demographic and episode-of-care factors, including those related to country of birth, socioeconomic status and marriage and insurance status, also contributed independently to involuntary status in our findings. The data suggests that non-clinical factors contribute to the decision to impose involuntary care, and that involuntary treatment might be imposed disproportionately on some groups, including people born overseas or with indicators of social disadvantage. There are numerous pathways by which these outcomes might be generated, including those related to the person, such as their trust in and willingness to engage with healthcare systems; those related to the clinician, including their assessments of the patient's function and safety in the community; and the service, including culturally appropriate treatment options.

Our findings are consistent with a previous large study of involuntary treatment in Ontario, Canada, which found that involuntary care was more common in people experiencing acute psychosis along with extreme agitation or suicidal ideation, as well as with recent police contact, although the strength of these associations was weaker than that found in our study.^16^

Large-scale ecological analyses of UK data^5,17^ have found that involuntary treatment was more common in areas with high levels of deprivation. A recent meta-analysis found that male gender, single marital status, unemployment, receiving welfare benefits, being diagnosed with a psychotic disorder or bipolar disorder and previous involuntary hospital admission all had moderate associations with involuntary care.^10^ However, many studies have limited sample sizes and high risk of bias.^10^

Our study adds to meta-analytic evidence because meta-regression of study level data cannot easily explore interaction or covariation between variables in individuals. Where similar factors were available for our analysis, the results are broadly consistent; although our analysis found homelessness associated with voluntary, rather than involuntary status, which may reflect our large data-set being able to control for collinearity with other factors such as socioeconomic status.

The finding that gender has only a weak effect on involuntary status in our final model is also unexpected. Our study included measures of diagnosis, aggression, comorbidity and disability: a lack of association with gender after controlling for these factors may suggest that these variables may mediate the greater risk of involuntary care in males.

The association between race and involuntary treatment has received much attention, with migrants often found to be at greater risk of involuntarily treatment than locally born people.^18^ A systematic review found that Black Caribbean, Black African and South Asian people had a moderately increased risk of involuntary hospital admission compared with people of White ethnicity;^18^ however, that review some included studies that may be confounded by not controlling for differences in diagnosis between groups. We found that speaking a language other than English or being born overseas was weakly associated with an increased likelihood of involuntary treatment, but not for patients born in primarily English-speaking countries (the UK, Ireland, the USA or Canada).

Debate remains as to the cause for the increased rate of detentions among Black and minority ethnic groups, and whether it is confounded by relatively higher rates of psychotic illnesses^19,20^ or by lower rates of voluntary treatment. Our finding that involuntary status was not uniformly more likely among people born overseas suggests that the issue is not simply related to the act of migration. These findings persisted after adjusting for diagnosis, clinical severity and comorbidity, although the effect size was weaker than that found in the recent meta-analysis.^18^ It is notable that Australia has a much higher proportion of overseas-born people in its population than the many countries in which the issue of involuntary status and race has been examined.^21^

### Limitations

Using routinely collected administrative data allows for a view of involuntary care across the entire NSW adult population, but such data-sets have inevitable limitations. Our data cannot show the full range of community support factors available to patients, from both the healthcare system and other social and community groups. Given that, arguably, the Mental Health Act is constructed such that a decision about whether or not a person can be made subject to involuntary treatment hinges primarily on the clinician's conclusion that no voluntary care pathway is reasonably available, the availability of such supports is often likely to be decisive.^22^

In addition, our data cannot capture many factors relating to the relationship between an individual, a healthcare service and their treating team that are likely to increase or moderate the risk of involuntary care. Factors such as service organisation, clinical leadership, supervision, cultural sensitivity and safety may all contribute to clinical choices about the use of involuntary care. More research is needed to understand how these issues may contribute to varying rates of involuntary care between services.

Routinely collected administrative variables, such as country of birth or language spoken at home, are imprecise measures of race and culture. However, understanding this individual variation is the first step to being able to understand any variation that may exist between different services and their approach to similar patients.

In this study, involuntary care has been defined by legal status codes recorded in hospital administrative systems. These inevitably include missing data and some coding errors. We excluded approximately 4% of episodes that had a missing or invalid legal status. These episodes appeared similar to episodes of voluntary care, and therefore may have slightly overestimated the rate of involuntary care in NSW.

The data available to our study also includes missing values for other predictor variables examined. Demographic measures (such as country of birth, language spoken at home, accommodation marital and employment status) are recorded by hospital administrative staff at admission, and may be inaccurate or not updated. Routinely collected diagnoses are likely to underestimate the degree of substance use and other comorbidities, although previous studies of NSW data have suggested that the degree of underestimation is only modest.^23^ Clinician-rated outcome measures (HoNOS, LSP) used to assess aggression and disability have high rates of missing data. Overall, missing clinical data is likely to weaken the strength of any associations found.

An important limitation is that our study did not have ethical approval to examine the relationship between Aboriginal and Torres Strait Islander status and involuntary treatment. Aboriginal and Torres Strait Islander people were included in our data, but not separately identified. Aboriginal and Torres Strait Islander people report higher levels of distress than other Australians,^24^ are twice as likely to be admitted for mental healthcare and report less positive experiences of care.^25,26^ Further work is required to understand patterns and risks of involuntary care in Aboriginal and Torres Strait Islander Australians.

In summary, involuntary treatment in NSW has increased despite strategic plans to decrease its use,^27^ and this research indicates targeted interventions toward specific patient groups may be a promising area for development of interventions. The study identifies a number of key factors relating to patient presentation and diagnosis that are associated with involuntary status, as well as a number of variables including overseas country of birth, marital status and housing situation. More research is needed to understand the cause of these associations, and what contribution service-level variation makes toward in the use of involuntary treatment.

## Supporting information

Corderoy et al. supplementary material 1 Corderoy et al. supplementary material

Corderoy et al. supplementary material 2 Corderoy et al. supplementary material

Corderoy et al. supplementary material 3 Corderoy et al. supplementary material

## Data Availability

No data are available. Access to NSW Health data is available to researchers only with the specific approval of the New South Wales Population and Health Services Research Ethics Committee. That approval does not permit sharing of unit record data with other researchers.
