# Supplementary material for: Factors associated with involuntary mental healthcare in New South Wales, Australia
Source: BJPsych Open. 2024 Mar 4;10(2):e59. doi: 10.1192/bjo.2023.628 (PMC10951846; doi:10.1192/bjo.2023.628)
Supplement: Corderoy et al. supplementary material 1 — Corderoy et al. supplementary material [file S2056472423006282sup001.docx]

Supplementary Table 1: Mapping of NSW Health legal status codes to voluntary/involuntary status

| Legal status group (1) | Current Code (2) | Codeset (3) | Code (4) | Text (original codeset) |  |
| --- | --- | --- | --- | --- | --- |
| Invol | 23 | v5 | 23 | 23 Inebriates Act s.3(1) Order for Control of Inebriates |  |
| Invol | 25 | v5 | 25 | 25 Other State Mental Health Act |  |
| Invol | 30 | v5 | 30 | 30 Bail Act s.36A(2) Additional Bail conditions |  |
| Invol | 40 | v5 | 40 | 40 Children and Young Persons (Care and Protection) Act s.17 Director-General's request for assistance |  |
| Invol | 41 | v5 | 41 | 41 Children and Young Persons (Care and Protection) Act s.18 Obligation to co-operate |  |
| Invol | 42 | v5 | 42 | 42 Children and Young Persons (Care and Protection) Act s.53 Making of assessment orders |  |
| Invol | 43 | v5 | 43 | 43 Children and Young Persons (Care and Protection) Act s.74 Order for provision of support services |  |
| Invol | 44 | v5 | 44 | 44 Children and Young Persons (Care and Protection) Act s.75 Ord. to att. therapeutic or treatment prog |  |
| Invol | 45 | v5 | 45 | 45 Children and Young Persons (Care and Protection) Act s.123 - Compulsory Assistance |  |
| Invol | 46 | v5 | 46 | 46 Children and Young Persons (Care and Protection) Act s.132 Emergency compulsory assistance |  |
| Invol | 47 | v5 | 47 | 47 Children and Young Persons (Care and Protection) Act s.173 Med exam of children in need of care &protection |  |
| Invol | 48 | v5 | 48 | 48 Children and Young Persons (Care and Protection) Act s.174 Emergency medical treatment |  |
| Invol | 49 | v5 | 49 | 49 Children and Young Persons (Care and Protection) Act s.175 Special medical treatment |  |
| Invol | 60 | v5 | 60 | 60 Guardianship Act s.40 Consents Given By Persons Responsible For Patients |  |
| Invol | 61 | v5 | 61 | 61 Guardianship Act s.44 Tribunal May Give Consent |  |
| Invol | 62 | v5 | 62 | 62 Guardianship Act s.45AA Tribunal May Approve Clinical Trials |  |
| Invol | 63 | v5 | 63 | 63 Guardianship Act s.46A Power of Guardian to Override Patient's Objection to Treatment When Authorise |  |
| Invol | 70 | v5 | 70 | 70 Public Health Act s.4 Orders and Directions During State of Emergency |  |
| Invol | 71 | v5 | 71 | 71 Public Health Act s.22 Power to Require Medical Examination |  |
| Invol | 72 | v5 | 72 | 72 Public Health Act s.23 Making of a Public Health Order |  |
| Invol | 80 | v4 | 2A | 2A Inpatient: Detention on certificate of medical practitioner or accredited person |  |
| Invol | 80 | v5 | 80 | 80 Drug and Alcohol Treatment Act 2007- Involuntary |  |
| Invol | 1D | v5 | 1D | 1D Inpatient: Correctional patient |  |
| Invol | 1E | v5 | 1E | 1E Inpatient: Re-classification of involuntary patient as correctional patient |  |
| Invol | 2B | v5 | 2B | 2B Inpatient: Detention on information of ambulance officer |  |
| Invol | 2C | v3 | 6 | 06 Mental Health Act s.24 Involuntary - Police |  |
| Invol | 2C | v5 | 2C | 2C Inpatient: Detention after apprehension by police |  |
| Invol | 2D | v3 | 4 | 04 Mental Health Act s.21 Involuntary - Medical Practitioner |  |
| Invol | 2D | v5 | 2D | 2D Inpatient: Detention after order for medical examination or observation |  |
| Invol | 2E | v3 | 7 | 07 Mental Health Act s.25 & Mental Health (Criminal Procedure) s.33 Involuntary - Court Order |  |
| Invol | 2E | v5 | 2E | 2E Inpatient: Detention on order of Magistrate or bail officer |  |
| Invol | 2F | v3 | 11 | 11 Mental Health Act s.51 Temporary Involuntary Detention |  |
| Invol | 2F | v3 | 12 | 12 Mental Health Act s.57(3)(a) Continued Involuntary |  |
| Invol | 2F | v3 | 13 | 13 Mental Health Act s.57(3)(b) Further Temporary Involuntary Detention |  |
| Invol | 2F | v3 | 14 | 14 Mental Health Act s.59(3) Reviewed Continued Involuntary |  |
| Invol | 2F | v5 | 2F | 2F Inpatient: Detention on order of the Mental Health Review Tribunal |  |
| Invol | 2G | v3 | 15 | 15 Mental Health Act s.78 Temporary or Continued Transfer |  |
| Invol | 2G | v5 | 2G | 2G Inpatient: Detention after transfer from another health facility |  |
| Invol | 2H | v3 | 5 | 05 Mental Health Act s.23 Involuntary - Relative/Friend |  |
| Invol | 2H | v5 | 2H | 2H Inpatient: Detention on request of primary carer, relative or friend |  |
| Invol | 2J | v3 | 8 | 08 Mental Health Act s.26 Involuntary - Welfare Officer |  |
| Invol | 2J | v5 | 2J | 2J Inpatient: Detention order or request from other specified authorised person or body |  |
| Invol | 2K | v3 | 9 | 09 Mental Health Act s.29 First Hospital Assessment - Mentally Ill |  |
| Invol | 2K | v5 | 2K | 2K Inpatient: Detention on certificate of medical practitioner or accredited person (Mentally Ill) |  |
| Invol | 2L | v3 | 10 | 10 Mental Health Act s.29 First Hospital Assessment - Mentally Disordered |  |
| Invol | 2L | v5 | 2L | 2L Inpatient: Detention on certificate of medical practitioner or accredited person (Mentally Disordere |  |
| Invol | 2M | v3 | 16 | 16 Mental Health Act s.118 Community Counselling Order |  |
| Invol | 2M | v3 | 17 | 17 Mental Health Act s.131 Community Treatment Order |  |
| Invol | 2M | v3 | 18 | 18 Mental Health Act s.143(1)(a) Breached Community Treatment Order - Mentally Ill |  |
| Invol | 2M | v3 | 19 | 19 Mental Health Act s.143(1)(b) Breached Community Treatment Order - Mentally Disordered |  |
| Invol | 2M | v3 | 20 | 20 Mental Health Act s.143A(3) Temporary Involuntary Post Community Treatment Order Breach |  |
| Invol | 2M | v5 | 2M | 2M Inpatient: Detention on breach of Community Treatment Order |  |
| Invol | 2N | v5 | 2N | 2N Inpatient: Detention with assistance of police on breach of Community Treatment Order |  |
| Invol | 4A | v3 | 22 | 22 Mental Health Act Schedule 1 Forensic |  |
| Invol | 4A | v5 | 4A | 4A Forensic patient |  |
| Invol | 4B | v3 | 21 | 21 Mental Health Act s.89 Forensic Reclassified As Continued |  |
| Invol | 4B | v5 | 4B | 4B Reclassification of forensic patient as involuntary patient |  |
| Invol | 4C | v5 | 4C | 4C Forensic Community Treatment Order |  |
| Vol | 98 | v5 | 98 | 98 No Act Applies |  |
| Vol | 1A | v3 | 1 | 01 Mental Health Act s.1(1) Informal |  |
| Vol | 1A | v5 | 1A | 1A Inpatient: Voluntary – Admission on own request |  |
| Vol | 1B | v3 | 2 | 02 Mental Health Act s.12(2) Informal Guardianship Order |  |
| Vol | 1B | v5 | 1B | 1B Inpatient: Voluntary admission of persons under guardianship |  |
| Vol | 1C | v5 | 1C | 1C Inpatient: Re-classification of involuntary patient as voluntary patient |  |
| Vol | 1F | v5 | 1F | 1F Inpatient: Re-classification of correctional patient as voluntary patient |  |
| Vol | 3A | v3 | 3 | 03 Mental Health Act s.135A Informal Community Treatment Order |  |
| Vol | 3A | v5 | 3A | 3A Ambulatory or inpatient: Community Treatment Order |  |
| Vol | 3B | v5 | 3B | 3B Ambulatory: Voluntary mental health client |  |
| Unk | 95 | v5 | 95 | 95 Other Legal Status |  |
| Unk | 99 | v5 | 99 | 99 Not Known / Not Stated |  |
| Unk | (Null) | - | - | (Null) |  |
| *(1) Invol = Involuntary, Vol = Voluntary, Unk = Unknown (2) Code as mapped to NSW legal status codes Version 5. (3) Codeset used at time of episode of care. (4) Code as recorded at time of episode of care.* | | | | |  |
|  |  |  |  |  |  |
