# Supplementary material for: Factors associated with involuntary mental healthcare in New South Wales, Australia
Source: BJPsych Open. 2024 Mar 4;10(2):e59. doi: 10.1192/bjo.2023.628 (PMC10951846; doi:10.1192/bjo.2023.628)
Supplement: Corderoy et al. supplementary material 2 — Corderoy et al. supplementary material [file S2056472423006282sup002.docx]

Supplementary Table: Characteristics of hospital admissions with missing legal status. Of 172,712 in-scope admissions to NSW mental health units, 6,606 (3.8%) did not have a valid legal status recorded and were excluded from analysis. Differences within categories compared via Chi-square test, using proportions in records with valid legal status as expected distribution.

| DOMAIN AND VARIABLE | Value | Has legal status  Number (%) | Missing legal status  Number (%) | Chi-Square Test |
| --- | --- | --- | --- | --- |
| PERSONAL |  |  |  |  |
| Sex | Male | 88,522 (53.3%) | 3,199 (48.4%) | X^2^ (1, N=172 630)=62.5, p<0.0001 |
|  | Female | 77,507 (46.7%) | 3,402 (51.5%) |  |
| Age group | 14-17 | 10,010 (6.0%) | 737 (11.2%) | X^2^ (7, N=172 712)=460.4, p<0.0001 |
|  | 18-24 | 27,324 (16.4%) | 1,245 (18.8%) |  |
|  | 25-35 | 38,895 (23.4%) | 1,412 (21.4%) |  |
|  | 35-45 | 36,841 (22.2%) | 1,251 (18.9%) |  |
|  | 45-55 | 28,719 (17.3%) | 959 (14.5%) |  |
|  | 55-65 | 14,690 (8.8%) | 489 (7.4%) |  |
|  | 65-75 | 6,535 (3.9%) | 311 (4.7%) |  |
|  | 75+ | 3,092 (1.9%) | 202 (3.1%) |  |
| Country of birth | Australia | 134,050 (80.7%) | 5,340 (80.8%) | X^2^ (6, N=172 712)=19.9, p=0.0029 |
|  | Asia | 10,408 (6.3%) | 366 (5.5%) |  |
|  | Africa, Middle East | 5,921 (3.6%) | 206 (3.1%) |  |
|  | UK and Ireland | 5,159 (3.1%) | 238 (3.6%) |  |
|  | NZ and Pacific | 4,634 (2.8%) | 187 (2.8%) |  |
|  | Europe | 3,929 (2.4%) | 172 (2.6%) |  |
|  | Other | 2,005 (1.2%) | 97 (1.5%) |  |
| Preferred language | English | 157,729 (95.0%) | 6,337 (95.9%) | X^2^ (1, N=172 712)=13.0, p=0.0003 |
|  | Other | 8,377 (5.0%) | 269 (4.1%) |  |
| Marital status | Not married | 30,134 (18.1%) | 1,160 (17.6%) | X^2^ (2, N=172 712)=2.6, p=0.2783 |
|  | Married | 132,426 (79.7%) | 5,316 (80.5%) |  |
| Homeless | No | 160,009 (96.3%) | 6,482 (98.1%) | X^2^ (1, N=172 712)=60.1, p<0.0001 |
|  | Yes | 6,097 (3.7%) | 124 (1.9%) |  |
| Disadvantage | Least (Quintile 1-3) | 89,416 (53.8%) | 2,974 (45.0%) | X^2^ (2, N=172 712)=288.3, p<0.0001 |
|  | Most (Quintile 4-5) | 60,400 (36.4%) | 3,065 (46.4%) |  |

| DOMAIN AND VARIABLE | Value | Has legal status  Number (%) | Missing legal status  Number (%) | Chi-Square Test |
| --- | --- | --- | --- | --- |
| CLINICAL |  |  |  |  |
| Diagnosis group | Affective | 32,018 (19.3%) | 1,290 (19.5%) | X^2^ (8, N=172 712)=420.1, p<0.0001 |
|  | Anx & Adj | 24,897 (15.0%) | 1,307 (19.8%) |  |
|  | Eating Dis | 653 (0.4%) | 25 (0.4%) |  |
|  | Inj & Poisoning | 18,988 (11.4%) | 1,024 (15.5%) |  |
|  | Non-MH | 1,155 (0.7%) | 30 (0.5%) |  |
|  | Organic MH | 14,517 (8.7%) | 691 (10.5%) |  |
|  | Other MH | 6,928 (4.2%) | 283 (4.3%) |  |
|  | Psychosis | 44,664 (26.9%) | 1,240 (18.8%) |  |
|  | Substance | 22,286 (13.4%) | 716 (10.8%) |  |
| Substances | None | 127,272 (76.6%) | 5,384 (81.5%) | X^2^ (3, N=0 276)=105.9, p<0.0001 |
|  | Cannabis only | 14,198 (8.5%) | 527 (8.0%) |  |
|  | Cannabis+Amphetamine | 9,887 (6.0%) | 284 (4.3%) |  |
|  | Amphetamine only | 14,749 (8.9%) | 411 (6.2%) |  |
| Personality disorder | No | 138,497 (83.4%) | 5,275 (79.9%) | X^2^ (1, N=0 393)=59.3, p<0.0001 |
|  | Yes | 27,609 (16.6%) | 1,331 (20.1%) |  |
| Developmental disability | No | 162,101 (97.6%) | 6,477 (98.0%) | X^2^ (-1, N=0 000)=0.0, p=0.0152 |
|  | Yes | 4,005 (2.4%) | 129 (2.0%) |  |
| Aggression (HoNOS) | No | 72,647 (43.7%) | 2,644 (40.0%) | X^2^ (2, N=6 447)=243.6, p<0.0001 |
|  | Yes | 31,600 (19.0%) | 925 (14.0%) |  |
|  | Unknown | 61,859 (37.2%) | 3,037 (46.0%) |  |
| Life Skills Profile | LSP Hi | 2,133 (1.3%) | 38 (0.6%) | X^2^ (2, N=0 000)=27.3, p<0.0001 |
|  | LSP Low | 1,713 (1.0%) | 60 (0.9%) |  |
|  | No LSP | 162,260 (97.7%) | 6,508 (98.5%) |  |

| DOMAIN AND VARIABLE | Value | Has legal status  Number (%) | Missing legal status  Number (%) | Chi-Square Test |
| --- | --- | --- | --- | --- |
| EPISODE OF CARE |  |  |  |  |
| Insurance status | None | 144,041 (86.7%) | 5,354 (81.0%) | X^2^ (2, N=0 068)=192.7, p<0.0001 |
|  | Private | 11,723 (7.1%) | 708 (10.7%) |  |
|  | Unknown | 10,342 (6.2%) | 544 (8.2%) |  |
| Source of referral | CHC & Outpatient | 16,409 (9.9%) | 383 (5.8%) | X^2^ (6, N=0 411)=856.0, p<0.0001 |
|  | Crisis team | 4,651 (2.8%) | 41 (0.6%) |  |
|  | ED | 81,507 (49.1%) | 4,237 (64.1%) |  |
|  | Legal | 5,943 (3.6%) | 141 (2.1%) |  |
|  | Other Hospital | 35,416 (21.3%) | 1,413 (21.4%) |  |
|  | Self or family | 8,196 (4.9%) | 89 (1.3%) |  |
|  | Unknown & Other | 13,984 (8.4%) | 302 (4.6%) |  |
| Catchment group | Interstate | 3,411 (2.1%) | 103 (1.6%) | X^2^ (4, N=1 408)=77.3, p<0.0001 |
|  | Other LHD | 30,654 (18.5%) | 1,017 (15.4%) |  |
|  | Same LHD | 30,654 (18.5%) | 1,017 (15.4%) |  |
|  | Same catchment | 21,540 (13.0%) | 807 (12.2%) |  |
| Length of stay | 0 (Same day) | 11,776 (7.1%) | 676 (10.2%) | X^2^ (8, N=6 507)=1232.8, p<0.0001 |
|  | 1 day | 22,614 (13.6%) | 1,451 (22.0%) |  |
|  | 2-3 days | 31,371 (18.9%) | 1,737 (26.3%) |  |
|  | 4-7 days | 33,616 (20.2%) | 1,316 (19.9%) |  |
|  | 8-14 days | 26,166 (15.8%) | 685 (10.4%) |  |
|  | 15-30 days | 23,249 (14.0%) | 476 (7.2%) |  |
|  | 31-90 days | 14,591 (8.8%) | 216 (3.3%) |  |
|  | 91-180 days | 1,559 (0.9%) | 12 (0.2%) |  |
|  | 181 days plus | 1,164 (0.7%) | 37 (0.6%) |  |
