# Supplementary material for: Factors associated with involuntary mental healthcare in New South Wales, Australia
Source: BJPsych Open. 2024 Mar 4;10(2):e59. doi: 10.1192/bjo.2023.628 (PMC10951846; doi:10.1192/bjo.2023.628)
Supplement: Corderoy et al. supplementary material 3 — Corderoy et al. supplementary material [file S2056472423006282sup003.docx]

Supplementary Table 3a: Sensitivity analysis excluding COVID period (2020, 2021). Cohort numbers and characteristics.

|  |  | NUMBERS |  | INVOLUNTARY % | |  |
| --- | --- | --- | --- | --- | --- | --- |
|  |  | N | % | Invol | Vol | Invol % |
| TOTAL |  |  |  |  |  |  |
| Episodes of care |  | 121,930 | 100% | 65,271 | 56,659 | 54% |
| PERSON VARIABLES |  |  |  |  |  |  |
| Sex | M | 65,430 | 54% | 37,276 | 28,154 | 57% |
|  | F | 56,464 | 46% | 27,986 | 28,478 | 50% |
|  | Other | 36 | 0% | 9 | 27 | 25% |
| Age group | 14-17 | 7,068 | 6% | 2,650 | 4,418 | 37% |
|  | 18-24 | 19,653 | 16% | 10,319 | 9,334 | 53% |
|  | 25-35 | 28,688 | 24% | 16,455 | 12,233 | 57% |
|  | 35-45 | 27,611 | 23% | 15,561 | 12,050 | 56% |
|  | 45-55 | 21,057 | 17% | 11,325 | 9,732 | 54% |
|  | 55-65 | 10,883 | 9% | 5,822 | 5,061 | 53% |
|  | 65-75 | 4,734 | 4% | 2,233 | 2,501 | 47% |
|  | 75+ | 2,238 | 2% | 907 | 1,331 | 41% |
| Country of birth | Africa & Middle East | 4,403 | 4% | 2,642 | 1,761 | 60% |
|  | Asia | 7,819 | 6% | 4,747 | 3,072 | 61% |
|  | Australia | 97,762 | 80% | 51,461 | 46,301 | 53% |
|  | Europe | 3,053 | 3% | 1,696 | 1,357 | 56% |
|  | NZ & Pacific | 3,471 | 3% | 2,039 | 1,432 | 59% |
|  | Other or unknown | 1,561 | 1% | 901 | 660 | 58% |
|  | UK Ireland US Canada | 3,863 | 3% | 1,786 | 2,077 | 46% |
| Language | Not English | 6,367 | 5% | 3,885 | 2,482 | 61% |
|  | English | 115,565 | 95% | 61,387 | 54,178 | 53% |
| Marital status | Married | 22,402 | 18% | 10,905 | 11,497 | 49% |
|  | Unknown | 2,529 | 2% | 1,767 | 762 | 70% |
|  | Not married | 97,001 | 80% | 52,600 | 44,401 | 54% |
| Employment | Employed | 2,226 | 2% | 788 | 1,438 | 35% |
|  | Student | 1,494 | 1% | 434 | 1,060 | 29% |
|  | Unknown | 108,970 | 89% | 59,227 | 49,743 | 54% |
|  | Not employed | 9,242 | 8% | 4,823 | 4,419 | 52% |
| Homeless | Yes | 4,508 | 4% | 2,498 | 2,010 | 55% |
|  | No | 117,424 | 96% | 62,774 | 54,650 | 53% |
| Disadvantage | Least (Quintile 1-3) | 65,889 | 54% | 33,503 | 32,386 | 51% |
|  | Most (Quintile 4-5) | 43,860 | 36% | 24,757 | 19,103 | 56% |
|  | Unknown | 12,183 | 10% | 7,012 | 5,171 | 58% |
| Rurality | Major cities | 57,449 | 47% | 30,283 | 27,166 | 53% |
|  | Inner regional | 38,817 | 32% | 20,850 | 17,967 | 54% |
|  | Outer and remote | 13,483 | 11% | 7,127 | 6,356 | 53% |
|  | Unknown | 12,183 | 10% | 7,012 | 5,171 | 58% |
| CLINICAL AND DIAGNOSTIC VARIABLES | |  |  |  |  |  |
| Primary diagnosis | Affective | 24,697 | 20% | 11,851 | 12,846 | 48% |
|  | Anx & Adj | 18,083 | 15% | 6,081 | 12,002 | 34% |
|  | Eating Dis | 462 | 0% | 198 | 264 | 43% |
|  | Inj & Poisoning | 14,161 | 12% | 5,932 | 8,229 | 42% |
|  | Non-MH | 3,443 | 3% | 1,635 | 1,808 | 47% |
|  | Organic MH | 871 | 1% | 593 | 278 | 68% |
|  | Other MH | 10,383 | 9% | 4,645 | 5,738 | 45% |
|  | Psychosis | 33,632 | 28% | 23,832 | 9,800 | 71% |
|  | Substance | 16,200 | 13% | 10,505 | 5,695 | 65% |
| Comorbidity | Alcohol | 19,983 | 16% | 10,497 | 9,486 | 53% |
|  | Cannabis | 17,452 | 14% | 11,592 | 5,860 | 66% |
|  | Amphetamines | 18,123 | 15% | 12,445 | 5,678 | 69% |
|  | *No Cann or amph* | *93,533* | *77%* | *46,311* | *47,222* | *50%* |
|  | *Cannabis only* | *10,276* | *8%* | *6,516* | *3,760* | *63%* |
|  | *Cannabis + Amphet* | *7,176* | *6%* | *5,076* | *2,100* | *71%* |
|  | *Amphetamine only* | *10,947* | *9%* | *7,369* | *3,578* | *67%* |
|  | Personality Disorder | 20,077 | 16% | 9,468 | 10,609 | 47% |
|  | Intellectual Disability | 3,016 | 2% | 1,727 | 1,289 | 57% |
| Aggression (HoNOS) | No | 53,394 | 44% | 26,475 | 26,919 | 50% |
|  | Yes | 22,555 | 18% | 17,020 | 5,535 | 75% |
|  | Unknown | 45,983 | 38% | 21,777 | 24,206 | 47% |
| Self-harm (HoNOS) | No | 51,993 | 43% | 31,294 | 20,699 | 60% |
|  | Yes | 23,956 | 20% | 12,201 | 11,755 | 51% |
|  | Unknown | 45,983 | 38% | 21,777 | 24,206 | 47% |
| HoNOS Score | No Honos | 45,983 | 38% | 21,777 | 24,206 | 47% |
|  | Q1 (Low) | 31,835 | 26% | 16,426 | 15,409 | 52% |
|  | Q2 | 16,612 | 14% | 9,535 | 7,077 | 57% |
|  | Q3 | 13,699 | 11% | 8,338 | 5,361 | 61% |
|  | Q4 (Hi) | 13,803 | 11% | 9,196 | 4,607 | 67% |
| LSP | LSP Hi | 1,557 | 1% | 1,155 | 402 | 74% |
|  | LSP Low | 1,220 | 1% | 580 | 640 | 48% |
|  | No LSP | 119,155 | 98% | 63,537 | 55,618 | 53% |
|  |  |  |  |  |  |  |
| EPISODE OF CARE |  |  |  |  |  |  |
| Insurance status | None | 105,649 | 87% | 57,052 | 48,597 | 54% |
|  | Private | 8,664 | 7% | 3,916 | 4,748 | 45% |
|  | Unknown | 7,619 | 6% | 4,304 | 3,315 | 56% |
| Source of referral | CHC & Outpatient | 12,132 | 10% | 4,519 | 7,613 | 37% |
|  | Crisis team | 3,588 | 3% | 2,035 | 1,553 | 57% |
|  | ED | 60,753 | 50% | 31,761 | 28,992 | 52% |
|  | Legal | 4,535 | 4% | 3,861 | 674 | 85% |
|  | Other Hospital | 25,495 | 21% | 16,467 | 9,028 | 65% |
|  | Self or family | 6,150 | 5% | 1,324 | 4,826 | 22% |
|  | Unknown & Other | 9,279 | 8% | 5,305 | 3,974 | 57% |
| Medicare eligible | No | 1,399 | 1% | 880 | 519 | 63% |
|  | Yes | 120,533 | 99% | 64,392 | 56,141 | 53% |
| Emergency status | Emergency | 102,478 | 84% | 54,900 | 47,578 | 54% |
|  | Other/Unknown | 11,558 | 9% | 7,054 | 4,504 | 61% |
|  | Planned | 7,896 | 6% | 3,318 | 4,578 | 42% |
| Catchment group | Interstate | 2,692 | 2% | 1,688 | 1,004 | 63% |
|  | NFA/Unk | 9,491 | 8% | 5,324 | 4,167 | 56% |
|  | Other LHD | 21,465 | 18% | 11,110 | 10,355 | 52% |
|  | Same LHD | 15,794 | 13% | 8,568 | 7,226 | 54% |
|  | Same catchment | 72,490 | 59% | 38,582 | 33,908 | 53% |
| Voluntary unit | No | 120,042 | 98% | 65,236 | 54,806 | 54% |
|  | Yes | 1,890 | 2% | 36 | 1,854 | 2% |

Supplementary Table 3b: Sensitivity analysis excluding COVID period (2020, 2021). Associations between involuntary care and variables in the personal, illness and episode of care domains, using binary logistic regression for individual variables and multivariable logistic regression within each domain.

|  |  | BIVARIATE | |  | MULTIVARIABLE WITHIN DOMAIN | |
| --- | --- | --- | --- | --- | --- | --- |
|  |  | OR | (95%CI) sig | AUC | OR | (95%CI) sig |
| TOTAL |  |  |  |  |  |  |
| Episodes of care |  |  |  |  |  |  |
| PERSON VARIABLES |  |  |  |  |  |  |
| Sex | M | 1.00 | - | 0.538 | 1.00 | - |
|  | F | 0.74 | (0.73-0.76)*** |  | 0.79 | (0.77-0.80)*** |
|  | Other | 0.25 | (0.12-0.54)*** |  | 0.29 | (0.14-0.63)** |
| Age group | 14-17 | 0.46 | (0.44-0.49)*** | 0.548 | 0.49 | (0.46-0.51)*** |
|  | 18-24 | 0.86 | (0.83-0.89)*** |  | 0.85 | (0.82-0.88)*** |
|  | 25-35 | 1.04 | (1.01-1.08)* |  | 1.02 | (0.99-1.06) |
|  | 35-45 | 1.00 | - |  | 1.00 | - |
|  | 45-55 | 0.90 | (0.87-0.93)*** |  | 0.92 | (0.89-0.95)*** |
|  | 55-65 | 0.89 | (0.85-0.93)*** |  | 0.91 | (0.87-0.95)*** |
|  | 65-75 | 0.69 | (0.65-0.74)*** |  | 0.74 | (0.70-0.79)*** |
|  | 75+ | 0.53 | (0.48-0.58)*** |  | 0.58 | (0.53-0.64)*** |
| Country of birth | Africa & Middle East | 1.35 | (1.27-1.44)*** | 0.523 | 1.25 | (1.17-1.33)*** |
|  | Asia | 1.39 | (1.33-1.46)*** |  | 1.35 | (1.28-1.43)*** |
|  | Australia | 1.00 | - |  | 1.00 | - |
|  | Europe | 1.12 | (1.05-1.21)** |  | 1.20 | (1.12-1.30)*** |
|  | NZ & Pacific | 1.28 | (1.20-1.37)*** |  | 1.25 | (1.16-1.33)*** |
|  | Other or unknown | 1.23 | (1.11-1.36)*** |  | 1.11 | (1.00-1.23) |
|  | UK Ireland US Canada | 0.77 | (0.73-0.83)*** |  | 0.83 | (0.78-0.89)*** |
| Language | Not English | 1.38 | (1.31-1.45)*** | 0.508 | 1.14 | (1.07-1.21)*** |
|  | English | 1.00 | - |  | 1.00 | - |
| Marital status | Married | 1.00 | - | 0.522 | 1.00 | - |
|  | Unknown | 1.25 | (1.21-1.29)*** |  | 1.30 | (1.26-1.34)*** |
|  | Not married | 2.44 | (2.24-2.67)*** |  | 2.28 | (2.09-2.50)*** |
| Employment | Employed | 1.00 | - | 0.515 |  |  |
|  | Student | 0.75 | (0.65-0.86)*** |  |  |  |
|  | Unknown | 2.17 | (1.99-2.37)*** |  |  |  |
|  | Not employed | 1.99 | (1.81-2.19)*** |  |  |  |
| Homeless | Yes | 1.08 | (1.02-1.15)* | 0.502 | 0.97 | (0.91-1.03) |
|  | No | 1.00 | - |  | 1.00 | - |
| Disadvantage | Least (Quintile 1-3) | 1.00 | - | 0.526 | 1.00 | - |
|  | Most (Quintile 4-5) | 1.25 | (1.22-1.28)*** |  | 1.25 | (1.22-1.28)*** |
|  | Unknown | 1.31 | (1.26-1.36)*** |  | 1.17 | (1.12-1.21)*** |
| Rurality | Major cities | 1.00 | - | 0.511 |  |  |
|  | Inner regional | 1.04 | (1.01-1.07)** |  |  |  |
|  | Outer and remote | 1.01 | (0.97-1.04) |  |  |  |
|  | Unknown | 1.22 | (1.17-1.27)*** |  |  |  |
| CLINICAL AND DIAGNOSTIC VARIABLES | |  |  |  |  |  |
| Primary diagnosis | Affective | 1.82 | (1.75-1.89)*** | 0.658 | 1.80 | (1.73-1.87)*** |
|  | Anx & Adj | 1.00 | - |  | 1.00 | - |
|  | Eating Dis | 1.48 | (1.23-1.78)*** |  | 1.64 | (1.36-1.98)*** |
|  | Inj & Poisoning | 1.42 | (1.36-1.49)*** |  | 1.47 | (1.41-1.54)*** |
|  | Non-MH | 1.78 | (1.66-1.92)*** |  | 1.85 | (1.71-1.99)*** |
|  | Organic MH | 4.21 | (3.64-4.87)*** |  | 4.42 | (3.82-5.13)*** |
|  | Other MH | 1.60 | (1.52-1.68)*** |  | 1.58 | (1.49-1.67)*** |
|  | Psychosis | 4.80 | (4.62-4.99)*** |  | 4.18 | (4.02-4.35)*** |
|  | Substance | 3.64 | (3.48-3.81)*** |  | 2.52 | (2.40-2.65)*** |
| Comorbidity | Alcohol | 0.95 | (0.92-0.98)** | 0.504 | 0.00 |  |
|  | Cannabis | 1.87 | (1.81-1.94)*** | 0.538 |  |  |
|  | Amphetamines | 2.12 | (2.05-2.19)*** | 0.546 |  |  |
|  | *No Cann or amph* | 1.00 | - |  | 1.00 | - |
|  | *Cannabis only* | 1.77 | (1.69-1.84)*** |  | 1.53 | (1.47-1.60)*** |
|  | *Cannabis + Amphet* | 2.46 | (2.34-2.60)*** |  | 1.82 | (1.72-1.92)*** |
|  | *Amphetamine only* | 2.10 | (2.01-2.19)*** |  | 1.55 | (1.48-1.63)*** |
|  | Personality Disorder | 0.74 | (0.71-0.76)*** | 0.522 | 0.91 | (0.88-0.95)*** |
|  | Intellectual Disability | 1.17 | (1.09-1.26)*** | 0.501 | 1.25 | (1.15-1.35)*** |
| Aggression (HoNOS) | No | 1.00 | - | 0.589 | 1.00 | - |
|  | Yes | 3.13 | (3.02-3.24)*** |  | 2.65 | (2.55-2.75)*** |
|  | Unknown | 0.91 | (0.89-0.94)*** |  | 0.95 | (0.93-0.98)*** |
| Self-harm (HoNOS) | No | 1.00 | - | 0.562 |  |  |
|  | Yes | 0.69 | (0.67-0.71)*** |  |  |  |
|  | Unknown | 0.60 | (0.58-0.61)*** |  |  |  |
| HoNOS Score | No Honos | 1.00 | - | 0.570 |  |  |
|  | Q1 (Low) | 1.18 | (1.15-1.22)*** |  |  |  |
|  | Q2 | 1.50 | (1.45-1.55)*** |  |  |  |
|  | Q3 | 1.73 | (1.66-1.80)*** |  |  |  |
|  | Q4 (Hi) | 2.22 | (2.13-2.31)*** |  |  |  |
| LSP | LSP Hi | 3.17 | (2.70-3.72)*** | 0.506 | 2.26 | (1.91-2.68)*** |
|  | LSP Low | 1.00 | - |  | 1.00 | - |
|  | No LSP | 1.26 | (1.13-1.41)*** |  | 1.25 | (1.11-1.41)*** |
|  |  |  |  |  |  |  |
| EPISODE OF CARE |  |  |  |  |  |  |
| Insurance status | None | 1.00 | - | 0.522 | 1.00 | - |
|  | Private | 0.70 | (0.67-0.73)*** |  | 0.70 | (0.67-0.73)*** |
|  | Unknown | 1.11 | (1.06-1.16)*** |  | 1.06 | (1.01-1.12)* |
| Source of referral | CHC & Outpatient | 0.54 | (0.52-0.56)*** | 0.622 | 0.60 | (0.57-0.62)*** |
|  | Crisis team | 1.20 | (1.12-1.28)*** |  | 1.21 | (1.13-1.30)*** |
|  | ED | 1.00 | - |  | 1.00 | - |
|  | Legal | 5.23 | (4.81-5.68)*** |  | 5.13 | (4.71-5.57)*** |
|  | Other Hospital | 1.67 | (1.62-1.72)*** |  | 1.71 | (1.66-1.77)*** |
|  | Self or family | 0.25 | (0.24-0.27)*** |  | 0.25 | (0.23-0.27)*** |
|  | Unknown & Other | 1.22 | (1.17-1.27)*** |  | 1.25 | (1.20-1.31)*** |
| Medicare eligible | No | 1.48 | (1.33-1.65)*** | 0.501 |  |  |
|  | Yes | 1.00 | - |  | 1.00 | - |
| Emergency status | Emergency | 1.00 | - | 0.526 | 1.00 | - |
|  | Other/Unknown | 1.36 | (1.30-1.41)*** |  | 0.00 |  |
|  | Planned | 0.63 | (0.60-0.66)*** |  | 0.00 |  |
| Catchment group | Interstate | 1.48 | (1.36-1.60)*** | 0.518 | 1.42 | (1.30-1.54)*** |
|  | NFA/Unk | 1.12 | (1.08-1.17)*** |  | 1.03 | (0.98-1.08) |
|  | Other LHD | 0.94 | (0.91-0.97)*** |  | 0.92 | (0.89-0.95)*** |
|  | Same LHD | 1.04 | (1.01-1.08)* |  | 0.96 | (0.92-0.99)* |
|  | Same catchment | 1.00 | - |  |  |  |
| Voluntary unit | No | 1.00 | - |  |  |  |
|  | Yes | 0.02 | (0.01-0.02)*** |  | 0.02 | (0.01-0.02)*** |

Supplementary Table 3c: Sensitivity analysis excluding COVID period (2020, 2021). Associations between involuntary care and variables in the personal, illness and episode of care domains, using multivariable logistic regression across all domains.

|  |  | BIVARIATE | |  | MULTIVARIABLE WITHIN DOMAIN | |
| --- | --- | --- | --- | --- | --- | --- |
|  |  | OR | (95%CI) sig | AUC | OR | (95%CI) sig |
| TOTAL |  |  |  |  |  |  |
| Episodes of care |  |  |  |  |  |  |
| PERSON VARIABLES |  |  |  |  |  |  |
| Sex | M | 1.00 | - | 0.538 | 1.00 | - |
|  | F | 0.74 | (0.73-0.76)*** |  | 0.79 | (0.77-0.80)*** |
|  | Other | 0.25 | (0.12-0.54)*** |  | 0.29 | (0.14-0.63)** |
| Age group | 14-17 | 0.46 | (0.44-0.49)*** | 0.548 | 0.49 | (0.46-0.51)*** |
|  | 18-24 | 0.86 | (0.83-0.89)*** |  | 0.85 | (0.82-0.88)*** |
|  | 25-35 | 1.04 | (1.01-1.08)* |  | 1.02 | (0.99-1.06) |
|  | 35-45 | 1.00 | - |  | 1.00 | - |
|  | 45-55 | 0.90 | (0.87-0.93)*** |  | 0.92 | (0.89-0.95)*** |
|  | 55-65 | 0.89 | (0.85-0.93)*** |  | 0.91 | (0.87-0.95)*** |
|  | 65-75 | 0.69 | (0.65-0.74)*** |  | 0.74 | (0.70-0.79)*** |
|  | 75+ | 0.53 | (0.48-0.58)*** |  | 0.58 | (0.53-0.64)*** |
| Country of birth | Africa & Middle East | 1.35 | (1.27-1.44)*** | 0.523 | 1.25 | (1.17-1.33)*** |
|  | Asia | 1.39 | (1.33-1.46)*** |  | 1.35 | (1.28-1.43)*** |
|  | Australia | 1.00 | - |  | 1.00 | - |
|  | Europe | 1.12 | (1.05-1.21)** |  | 1.20 | (1.12-1.30)*** |
|  | NZ & Pacific | 1.28 | (1.20-1.37)*** |  | 1.25 | (1.16-1.33)*** |
|  | Other or unknown | 1.23 | (1.11-1.36)*** |  | 1.11 | (1.00-1.23) |
|  | UK Ireland US Canada | 0.77 | (0.73-0.83)*** |  | 0.83 | (0.78-0.89)*** |
| Language | Not English | 1.38 | (1.31-1.45)*** | 0.508 | 1.14 | (1.07-1.21)*** |
|  | English | 1.00 | - |  | 1.00 | - |
| Marital status | Married | 1.00 | - | 0.522 | 1.00 | - |
|  | Unknown | 1.25 | (1.21-1.29)*** |  | 1.30 | (1.26-1.34)*** |
|  | Not married | 2.44 | (2.24-2.67)*** |  | 2.28 | (2.09-2.50)*** |
| Employment | Employed | 1.00 | - | 0.515 |  |  |
|  | Student | 0.75 | (0.65-0.86)*** |  |  |  |
|  | Unknown | 2.17 | (1.99-2.37)*** |  |  |  |
|  | Not employed | 1.99 | (1.81-2.19)*** |  |  |  |
| Homeless | Yes | 1.08 | (1.02-1.15)* | 0.502 | 0.97 | (0.91-1.03) |
|  | No | 1.00 | - |  | 1.00 | - |
| Disadvantage | Least (Quintile 1-3) | 1.00 | - | 0.526 | 1.00 | - |
|  | Most (Quintile 4-5) | 1.25 | (1.22-1.28)*** |  | 1.25 | (1.22-1.28)*** |
|  | Unknown | 1.31 | (1.26-1.36)*** |  | 1.17 | (1.12-1.21)*** |
| Rurality | Major cities | 1.00 | - | 0.511 |  |  |
|  | Inner regional | 1.04 | (1.01-1.07)** |  |  |  |
|  | Outer and remote | 1.01 | (0.97-1.04) |  |  |  |
|  | Unknown | 1.22 | (1.17-1.27)*** |  |  |  |
| CLINICAL AND DIAGNOSTIC VARIABLES | |  |  |  |  |  |
| Primary diagnosis | Affective | 1.82 | (1.75-1.89)*** | 0.658 | 1.80 | (1.73-1.87)*** |
|  | Anx & Adj | 1.00 | - |  | 1.00 | - |
|  | Eating Dis | 1.48 | (1.23-1.78)*** |  | 1.64 | (1.36-1.98)*** |
|  | Inj & Poisoning | 1.42 | (1.36-1.49)*** |  | 1.47 | (1.41-1.54)*** |
|  | Non-MH | 1.78 | (1.66-1.92)*** |  | 1.85 | (1.71-1.99)*** |
|  | Organic MH | 4.21 | (3.64-4.87)*** |  | 4.42 | (3.82-5.13)*** |
|  | Other MH | 1.60 | (1.52-1.68)*** |  | 1.58 | (1.49-1.67)*** |
|  | Psychosis | 4.80 | (4.62-4.99)*** |  | 4.18 | (4.02-4.35)*** |
|  | Substance | 3.64 | (3.48-3.81)*** |  | 2.52 | (2.40-2.65)*** |
| Comorbidity | Alcohol | 0.95 | (0.92-0.98)** | 0.504 | 0.00 |  |
|  | Cannabis | 1.87 | (1.81-1.94)*** | 0.538 |  |  |
|  | Amphetamines | 2.12 | (2.05-2.19)*** | 0.546 |  |  |
|  | *No Cann or amph* | 1.00 | - |  | 1.00 | - |
|  | *Cannabis only* | 1.77 | (1.69-1.84)*** |  | 1.53 | (1.47-1.60)*** |
|  | *Cannabis + Amphet* | 2.46 | (2.34-2.60)*** |  | 1.82 | (1.72-1.92)*** |
|  | *Amphetamine only* | 2.10 | (2.01-2.19)*** |  | 1.55 | (1.48-1.63)*** |
|  | Personality Disorder | 0.74 | (0.71-0.76)*** | 0.522 | 0.91 | (0.88-0.95)*** |
|  | Intellectual Disability | 1.17 | (1.09-1.26)*** | 0.501 | 1.25 | (1.15-1.35)*** |
| Aggression (HoNOS) | No | 1.00 | - | 0.589 | 1.00 | - |
|  | Yes | 3.13 | (3.02-3.24)*** |  | 2.65 | (2.55-2.75)*** |
|  | Unknown | 0.91 | (0.89-0.94)*** |  | 0.95 | (0.93-0.98)*** |
| Self-harm (HoNOS) | No | 1.00 | - | 0.562 |  |  |
|  | Yes | 0.69 | (0.67-0.71)*** |  |  |  |
|  | Unknown | 0.60 | (0.58-0.61)*** |  |  |  |
| HoNOS Score | No Honos | 1.00 | - | 0.570 |  |  |
|  | Q1 (Low) | 1.18 | (1.15-1.22)*** |  |  |  |
|  | Q2 | 1.50 | (1.45-1.55)*** |  |  |  |
|  | Q3 | 1.73 | (1.66-1.80)*** |  |  |  |
|  | Q4 (Hi) | 2.22 | (2.13-2.31)*** |  |  |  |
| LSP | LSP Hi | 3.17 | (2.70-3.72)*** | 0.506 | 2.26 | (1.91-2.68)*** |
|  | LSP Low | 1.00 | - |  | 1.00 | - |
|  | No LSP | 1.26 | (1.13-1.41)*** |  | 1.25 | (1.11-1.41)*** |
|  |  |  |  |  |  |  |
| EPISODE OF CARE |  |  |  |  |  |  |
| Insurance status | None | 1.00 | - | 0.522 | 1.00 | - |
|  | Private | 0.70 | (0.67-0.73)*** |  | 0.70 | (0.67-0.73)*** |
|  | Unknown | 1.11 | (1.06-1.16)*** |  | 1.06 | (1.01-1.12)* |
| Source of referral | CHC & Outpatient | 0.54 | (0.52-0.56)*** | 0.622 | 0.60 | (0.57-0.62)*** |
|  | Crisis team | 1.20 | (1.12-1.28)*** |  | 1.21 | (1.13-1.30)*** |
|  | ED | 1.00 | - |  | 1.00 | - |
|  | Legal | 5.23 | (4.81-5.68)*** |  | 5.13 | (4.71-5.57)*** |
|  | Other Hospital | 1.67 | (1.62-1.72)*** |  | 1.71 | (1.66-1.77)*** |
|  | Self or family | 0.25 | (0.24-0.27)*** |  | 0.25 | (0.23-0.27)*** |
|  | Unknown & Other | 1.22 | (1.17-1.27)*** |  | 1.25 | (1.20-1.31)*** |
| Medicare eligible | No | 1.48 | (1.33-1.65)*** | 0.501 |  |  |
|  | Yes | 1.00 | - |  | 1.00 | - |
| Emergency status | Emergency | 1.00 | - | 0.526 | 1.00 | - |
|  | Other/Unknown | 1.36 | (1.30-1.41)*** |  | 0.00 |  |
|  | Planned | 0.63 | (0.60-0.66)*** |  | 0.00 |  |
| Catchment group | Interstate | 1.48 | (1.36-1.60)*** | 0.518 | 1.42 | (1.30-1.54)*** |
|  | NFA/Unk | 1.12 | (1.08-1.17)*** |  | 1.03 | (0.98-1.08) |
|  | Other LHD | 0.94 | (0.91-0.97)*** |  | 0.92 | (0.89-0.95)*** |
|  | Same LHD | 1.04 | (1.01-1.08)* |  | 0.96 | (0.92-0.99)* |
|  | Same catchment | 1.00 | - |  |  |  |
| Voluntary unit | No | 1.00 | - |  |  |  |
|  | Yes | 0.02 | (0.01-0.02)*** |  | 0.02 | (0.01-0.02)*** |
